# Supplementary material for: Development of a discrete choice experiment questionnaire to elicit preferences by pregnant women and policymakers for the expansion of non-invasive prenatal screening
Source: PLoS One. 2023 Jun 23;18(6):e0287653. doi: 10.1371/journal.pone.0287653 (PMC10289448; doi:10.1371/journal.pone.0287653)
Supplement: S1 Text — (DOCX) [file pone.0287653.s001.docx]

REVIEW

Nguyen *et al.*

**Discrete choice experiments in prenatal screening for fetal anomalies: A systematic review of the literature**

Hung Manh Nguyen^1^, Bounhome Soukkhaphone^1^, Jason Robert Guertin^1,2^, François Rousseau^1,3^, Daniel Reinharz^1*^

^1^ Université Laval, Québec, Canada

^2^ Centre de Recherche du CHU de Québec – Université Laval, Québec, Canada

^3^ Hôpital Saint-François d’Assise, Québec, Canada

*** Corresponding author**: Daniel Reinharz, Département de médecine sociale et préventive, Université Laval, Québec, Canada, e-mail: daniel.reinharz@fmed.ulaval.ca

**Abstract**

*Objective*

This review aimed to identify relevant attributes influencing decision-making on conditions to be screened in prenatal screening program.

*Data sources*

Six databases (PubMed, Embase, EconLit, Cochrane Library, Web of Science, ProQuest Dissertations and Theses Global) were explored by using Mesh terms and text words to target studies using DCE for prenatal tests for fetal conditions. The reference list of the retrieved studies was also scanned for any additional relevant publications not found in the search.

*Study selection*

Entire identified publications were screened following the inclusion criteria: having a target population consisting of pregnant women or women who are at child-bearing age, having received a DCE questionnaire on prenatal tests for fetal conditions, and having a focus on DCE attributes.

*Data extraction*

The ISPOR checklist items were used to both assess methodological quality of the retained documents and extracting data with one additional item.

*Data synthesis*

Of the 58 DCE publications found during the database search, 13 met our eligibility criteria. Studies were conducted in high-income countries. Representativeness of the samples and generalizability of the results were generally non-optimal. Differences in preferences for different levels of the attributes between woman and other stakeholders could be identified. DCE could also produce willingness-to-pay values.

*Conclusion*

DCE looks as a promising tool both to better take into consideration the perspectives of the different stakeholders in the process of decision making on prenatal test, and as a tool to produce willingness-to-pay values that might be used in economic evaluations of a new prenatal screening or diagnostic test.

**Key words:** prenatal care, prenatal screening, discrete choice experiment, systematic review

**Introduction**

In many health care jurisdictions, deploying efforts to involve stakeholders into the decision process of defining what services should be offered in the public health care system is considered as desirable.^1^ Discrete choice experiment (DCE) is an economic approach increasingly used in order to give a voice to users of services into this process. DCE measurement proceeds by identifying the level of importance given by respondents, to a few attributes, for example waiting time or cost, considered as relevant for the decision to provide or not the technology under evaluation.^2–4^

The DCE approach could be particularly valuable in prenatal screening. Medical conditions that can be screened in prenatal screening program are numerous. Each condition that can be tested for is associated with its peculiar and complex ethical issues. A choice has therefore to be made. Ideally, the choice should be based on the medical impacts and clinical performance of a test, its cost and the social desirability of adding a particular condition to be tested among those included in currently implemented screening programs.

DCE in prenatal testing is a promising approach to measure the social desirability of a new test offer, as it can be used to identify what is important for key stakeholders, as health professionals, decision makers and parents, allowing the decision making process to adopt a social perspective.^5–7^ Indeed, this has been confirmed by a recent publication.^8^ This review of literature was therefore conducted to identify relevant attributes influencing decision-making on conditions to be screened in prenatal screening program.

**Methods**

This review of literature was conducted according to the Preferred Reporting Items for Systematic Reviews and Meta-Analyses Protocols 2015 (PRISMA-P 2015) recommendations.^9,10^

**Eligible criteria**

Studies were eligible if they used DCE to elicit preferences on prenatal screening and diagnosis tests for medical anomalies affecting the fetus. The articles or other documents had to be complete and written in English. There was no restriction of geographical areas applied.

**Search strategy**

We identified studies by broadly searching PubMed, EMBASE, EconLit, Cochrane Library and Web of Science databases from their inception to February 2019. The reference list of the retrieved studies was scanned for any additional relevant publications not found in the search. International dissertations and theses were also screened by searching ProQuest Dissertations and Theses Global to explore the presence of documents related to DCE in prenatal care. Search terms were used to target studies using DCE based on the PICO approach (see table 1): ‘discrete choice experiment’, ‘discrete choice experiments’, ‘discrete choice model’, ‘discrete choice models’, ‘discrete choice modelings’, ‘discrete choice conjoint experiment’, ‘path-worth utilities’, ‘functional measurement’, ‘paired comparison’, ‘pairwise choices’, ‘conjoint analysis’, ‘conjoint measurement’, ‘conjoint study’, ‘conjoint studies’, ‘conjoint choice experiment’, ‘conjoint choice experiments’ and ‘stated preference’. As suggested by Soekhai *et al,* we excluded the search terms “DCE” and “conjoint” since they were reported to yield too many irrelevant results.^11^ Medical Subject Heading (Mesh) terms (i.e. prenatal diagnosis, prenatal care, fetus, fetal disease) and text words were used for screening the population with the fetal condition. Attribute terms and their valuation were also used to target DCE conducted in prenatal care.

**Selection of studies**

The entire documents were screened independently by two reviewers (M.H.N and B.S) for the following inclusion criteria: having a target population consisting of pregnant women or women who are at child-bearing age, having received a DCE questionnaire on prenatal tests for fetal conditions, and having a focus on DCE attributes. Statistical significance of the agreement for the full-text eligibility was defined by using Kappa statistic. A consensus between the two reviewers was considered if the Kappa value ≥ 0.7. The reference list of the eligible studies was scanned for any relevant publications that were not found in the search. Any disagreement regarding the selection of an article was discussed until a consensus was reached. A third reviewer (D.R), if necessary, was implicated.

**Data extraction**

Data for each retained study were independently extracted and summarized by the two reviewers (H.M.N and B.S) on Excel spreadsheet. The extracted data related to the items of the ISPOR checklist (i.e., research questions, attributes and levels, construction of tasks, experimental design, preference elicitation, instrument design, data collection, statistical analysis, results, conclusions, and study presentation), to which one additional item was added: main findings.^12^ Any disagreement was solved by discussion and debate. The consensus search involved the third reviewer (D.R).

**Validity assessment**

A few grids are suggested to be used to verify methodological quality of DCE studies. The ISPOR checklist was used to assess the retained documents.^12^ As the review of literature aimed primarily at identifying the attributes considered in DCE studies, we considered the first nine ISPOR items and not the last one (i.e., study presentation). The checklist is not designed to produce a score to judge the quality of studies, but to highlight the coherence of the methodological design with good practice research.^12^ The assessment process was performed independently by the two reviewers (H.M.N and B.S). Any disagreement on the risk assessment of each study were solved by discussion between the two reviewers and a third reviewer (D.R).

**Data synthesis**

A thematic approach was used to synthesize the data of the retained studies. The ISPOR items were used as themes to identify attributes that have been considered in DCE studies in prenatal screening, preference results obtained from the DCEs, and preference heterogeneity among the studies, if applicable. Furthermore, no meta-analysis was performed in this review.

**Results**

**Characteristics of included DCE studies**

A total 13 studies were included in our review of literature (see figure 1). No study has been conducted with policymakers, i.e., voting members of Health Technology Assessment agencies committees mandated to make recommendations to the decision-maker, usually the Minister of Health, on prenatal screening policies. All were of high quality, i.e., all the ISPOR checklist items were reported, although the sample of participants enrolled in the study varied in terms of population representativeness. For example, the study population had an above-average individual income in Hong Kong and an above-average family income in the Netherlands.^13,14^ The representativeness of the location site, hospital or antennal centers/units could not always be ascertained. The population tented to be highly educated in Hong Kong, the UK and Denmark.^5,13,14^ An English version of the questionnaire was administrated to women in Singapore even if their first language was not English.^15^ The questionnaires were administrated to English-speaking groups of the population in the UK and Australia.^6,7,16^ A study was focused on pregnant women with low-risk pregnancies in the UK.^5^

Studies were conducted in high-income countries and in one territory (Hong Kong). All these countries have a sort of universal health coverage. Yet, these jurisdictions vary in terms of patients' financial contribution to prenatal care.

**Attribute identification and DCE preference results**

A summary of attribute identification in each DCE studies is presented in Table 2. The first studies were conducted in the first decade of the century.^17^ Most were concerned with defining attributes associated with women's decision to accept a prenatal screening test for Down syndrome. The arrival of non-invasive prenatal screening (NIPS) tests allowing detecting fetal cell-free DNA in maternal blood, reducing thereby the use of invasive prenatal procedures, seem to have given a boost to the use of DCE in studies on prenatal care. All studies on NIPS took place between 2012 and 2018. Willingness-to-pay for a change in attributes level, notably the amount of information provided to women and the time to wait to get test results, was measured in three studies.^13,18,19^ Two studies provided a valued benefit of receiving a prenatal test but no full cost-benefit study had been performed.^13,19^

While no single attribute was present in all questionnaires, some attributes appeared quite frequently (see table 2), notably the detection rate (11 studies), the risk of miscarriage (9 studies), the information content (7 studies) and the time of receiving results during a pregnancy (6 studies). Five articles had a cost attribute. Dimensions reflected the trade-off expected between options presented to respondents in the studies.

The DCE approach allowed identifying differences in preferences for attributes between subgroups of women based on age, country, ethnicity, presence of disease, income or personal experience with an obstetrical issue, as infertility treatment or a miscarriage.^5,6,13–15,20,21^ For example, women who were at an advanced maternal age, who had a previous miscarriage or knew a child with affected anomalies were found to have a higher preference for a test carrying lower risk of miscarriage. Cost and waiting time attributes were found to be associated with family income.^13^

DCE appeared also to be an effective method to distinguish groups of stakeholders. Studies were able to show that women and health professionals vary regarding the importance given to DCE attributes. Women tended to highly value the risk of miscarriage and the reception of comprehensive information, while health professionals tended to place higher value on detection rate of the test, the moment during pregnancy when test results would become available and the capacity to diagnose a condition earlier on.

**Discussion**

DCE looks as a promising avenue take into consideration the perspective of the population/patients in the use of a prenatal screening/diagnosis test. Results from studies considered in the review of literature effectively revealed what would have been expected from pregnant women. Consistent with the review of Vass *et al* (2019), women wanted to be informed and they were primarily concerned by the risk of miscarriage caused by the test procedure.^5,15,21,22^ The preoccupation for a miscarriage is consistent with general knowledge about the probability that a positive test might lead to an amniocentesis, which carries a risk of miscarriage. Most health care systems have also taken the journey towards implanting shared decision-making. Increasingly patients are incited to participate to the medical decision process. Health professional are increasingly expected to use available tools developed to inform patients in order to help them to choose among options.^5,6,15,18,26^ Patients have a role now in the care process that requires them to be informed. This is becoming the norm. DCE looks therefore as a relevant tool, as it allows measuring aspects of the decision-making process that are relevant from a pregnant woman perspective.

Moreover, studies showed that a DCE approach can identify, among the attributes considered, differences between women and other stakeholders, notably health care professionals. Most women favored a non-invasive procedure rather than an invasive one regarding the diagnosis of Down syndrome.^27,28^ Besides, women tended to be looking for comprehensive information on the risk that their baby might have a Down syndrome, suffer from a debilitating disease, or have a life-threatening condition or a short life span.^29^ It has even been shown that some women and their partners are willing to accept a small probability of miscarriage from an invasive amniocentesis if this is compensated by more information on a health risk for the baby.^27,29^ As for health professionals, they were more focused on the benefit of new technologies developed to offer benefits as an earlier screening or less invasive test, highlighting psychological added-values for women.^26,30^ Results for DCE studies are therefore consistent with what is known regarding preoccupations by women and by health professionals.

DCE is definitively an instrument that allows producing data on the value of prenatal testing attributed by pregnant women in contrast to the value attributed by health care professionals. The instrument is therefore of interest in further studies that might explore additional questions, notably the value of adding a new test into the list of tests included in a screening program. Considering the numerous medical, economic, and ethical issues that are associated with the development of screening tests, particularly when these test aim at genetic testing, better exploring the preferences by all stakeholders looks highly desirable. DCE is a promising avenue to make the decisions on prenatal testing accountable to the society.

Last, it is worth noting that the present review is only applicable for the identification of attributes that have been used in DCE studies for prenatal screening. This would limit the generalization of the review’s result in other topics. Furthermore, we did not perform meta-analysis in this review, hence sensitivity and specificity of preference outcomes measured by DCE are not confirmed.

**Conclusion**

This review of literature highlights the relevance of DCE to determine differences among stakeholder preferences regarding prenatal screening and diagnosis tests. Studies that exploit the potential of DCE-provided information for performing full cost-benefit studies, particularly studies that allow comparing women's preferences with decision makers' preferences, are therefore warranted.

**Fundings**

This study was funded through a grant from Genome Canada (with Génome Québec, Genome BC and Genome Alberta and le Ministère de l'économie, de l'innovation et des exportations (Gouvernement du Quebec) - Grant number: LSARP2012-4523), the Canadian Institutes for Health Research (Grant number: GPH129342).

**Disclosure**

The authors report no conflicts of interest in this work.

**Data availability**

Data extraction presented in Excel spreadsheet is available on request.

**References**

1. Mockford C, Staniszewska S, Griffiths F, Herron-Marx S. The impact of patient and public involvement on UK NHS health care: a systematic review. *International Journal for Quality in Health Care*. 2012;24(1):28-38. doi:10.1093/intqhc/mzr066

2. de Bekker-Grob EW, Ryan M, Gerard K. Discrete choice experiments in health economics: a review of the literature. *Health Economics*. 2012;21(2):145-172. doi:10.1002/hec.1697

3. Lancsar E, Louviere J. Conducting discrete choice experiments to inform healthcare decision making: A user’s guide. *PharmacoEconomics*. 2008;26(8):661-677. doi:10.2165/00019053-200826080-00004

4. Ryan M, Bate A, Eastmond CJ, Ludbrook A. Use of discrete choice experiments to elicit preferences. *Quality and Safety in Health Care*. 2001;10(Supplement 1):i55-i60. doi:10.1136/qhc.0100055

5. Hill M, Fisher J, Chitty LS, Morris S. Women’s and health professionals’ preferences for prenatal tests for Down syndrome: a discrete choice experiment to contrast noninvasive prenatal diagnosis with current invasive tests. *Genetics in Medicine*. 2012;14(11):905-913. doi:10.1038/gim.2012.68

6. Hill M, Suri R, Nash EF, Morris S, Chitty LS. Preferences for prenatal tests for cystic fibrosis: a discrete choice experiment to compare the views of adult patients, carriers of cystic fibrosis and health professionals. *J Clin Med*. 2014;3(1):176-190. doi:10.3390/jcm3010176

7. Lewis SM, Cullinane FN, Bishop AJ, Chitty LS, Marteau TM, Halliday JL. A comparison of Australian and UK obstetricians’ and midwives’ preferences for screening tests for Down syndrome. *Prenatal Diagnosis*. 2006;26(1):60-66. doi:10.1002/pd.1357

8. Vass CM, Georgsson S, Ulph F, Payne K. Preferences for aspects of antenatal and newborn screening: a systematic review. *BMC Pregnancy Childbirth*. 2019;19(1):131. doi:10.1186/s12884-019-2278-7

9. Moher D, Shamseer L, Clarke M, et al. Preferred reporting items for systematic review and meta-analysis protocols (PRISMA-P) 2015 statement. *Systematic Reviews*. 2015;4(1). doi:10.1186/2046-4053-4-1

10. Shamseer L, Moher D, Clarke M, et al. Preferred reporting items for systematic review and meta-analysis protocols (PRISMA-P) 2015: elaboration and explanation. *The British Medical Journal*. 2015;350(jan02 1):g7647-g7647. doi:10.1136/bmj.g7647

11. Soekhai V, de Bekker-Grob EW, Ellis AR, Vass CM. Discrete choice experiments in health economics: past, present and future. *PharmacoEconomics*. 2019;37(2):201-226. doi:10.1007/s40273-018-0734-2

12. Bridges JFP, Hauber AB, Marshall D, et al. Conjoint analysis applications in health - a checklist: a report of the ISPOR good research practices for conjoint analysis task force. *Value in Health*. 2011;14(4):403-413. doi:10.1016/j.jval.2010.11.013

13. Chan YM, Sahota DS, Leung TY, Choy KW, Chan OK, Lau TK. Chinese women’s preferences for prenatal diagnostic procedure and their willingness to trade between procedures. *Prenatal Diagnosis*. 2009;29(13):1270-1276. doi:10.1002/pd.2394

14. Lund ICB, Becher N, Petersen OB, Hill M, Chitty L, Vogel I. Preferences for prenatal testing among pregnant women, partners and health professionals. *Danish Medical Journal*. 2018;65(5):A5486.

15. Barrett AN, Advani HV, Chitty LS, et al. Evaluation of preferences of women and healthcare professionals in Singapore for implementation of noninvasive prenatal testing for Down syndrome. *Singapore Med J*. 2017;58(6):298-310. doi:10.11622/smedj.2016114

16. Carroll FE, Al-Janabi H, Flynn T, Montgomery AA. Women and their partners’ preferences for Down’s syndrome screening tests: a discrete choice experiment: Down’s syndrome DCE. *Prenatal Diagnosis*. 2013;33(5):449-456. doi:10.1002/pd.4086

17. Bishop AJ, Marteau TM, Armstrong D, et al. Women and health care professionals’ preferences for Down’s Syndrome screening tests: a conjoint analysis study. *BJOG: An International Journal of Obstetrics and Gynaecology*. 2004;111(8):775-779. doi:10.1111/j.1471-0528.2004.00197.x

18. Beulen L, Grutters JPC, Faas BHW, et al. Women’s and healthcare professionals’ preferences for prenatal testing: a discrete choice experiment. *Prenatal Diagnosis*. 2015;35(6):549-557. doi:10.1002/pd.4571

19. Ryan M, Diack J, Watson V, Smith N. Rapid prenatal diagnostic testing for Down syndrome only or longer wait for full karyotype: the views of pregnant women. *Prenat Diagn*. 2005;25(13):1206-1211. doi:10.1002/pd.1309

20. Hill M, Oteng-Ntim E, Forya F, Petrou M, Morris S, Chitty LS. Preferences for prenatal diagnosis of sickle-cell disorder: a discrete choice experiment comparing potential service users and health-care providers. *Health Expect*. 2017;20(6):1289-1295. doi:10.1111/hex.12568

21. Hill M, Johnson J-A, Langlois S, et al. Preferences for prenatal tests for Down syndrome: an international comparison of the views of pregnant women and health professionals. *Eur J Hum Genet*. 2016;24(7):968-975. doi:10.1038/ejhg.2015.249

22. Lewis SM, Cullinane FM, Carlin JB, Halliday JL. Women’s and health professionals’ preferences for prenatal testing for Down syndrome in Australia. *The Australian and New Zealand Journal of Obstetrics and Gynaecology*. 2006;46(3):205-211. doi:10.1111/j.1479-828X.2006.00567.x

23. Mulvey S, Zachariah R, McIlwaine K, Wallace EM. Do women prefer to have screening tests for Down syndrome that have the lowest screen-positive rate or the highest detection rate? *Prenat Diagn*. 2003;23(10):828-832. doi:10.1002/pd.701

24. Mujezinovic F, Alfirevic Z. Procedure-related complications of amniocentesis and chorionic villous sampling: a systematic review. *Obstetrics & Gynecology*. 2007;110(3):687-694. doi:10.1097/01.AOG.0000278820.54029.e3

25. Chan YM, Leung WC, Chan WP, Leung TY, Cheng YKY, Sahota DS. Women’s uptake of non-invasive DNA testing following a high-risk screening test for trisomy 21 within a publicly funded healthcare system: findings from a retrospective review: Chinese women’s uptake of NIDT after a high-risk Down syndrome screening test. *Prenat Diagn*. 2015;35(4):342-347. doi:10.1002/pd.4544

26. Yotsumoto J, Sekizawa A, Koide K, et al. Attitudes toward non-invasive prenatal diagnosis among pregnant women and health professionals in Japan: Attitudes toward NIPD. *Prenat Diagn*. 2012;32(7):674-679. doi:10.1002/pd.3886

27. Cheng Y, Leung W, Leung T, et al. Women’s preference for non-invasive prenatal DNA testing versus chromosomal microarray after screening for Down syndrome: a prospective study. *BJOG: Int J Obstet Gy*. 2018;125(4):451-459. doi:10.1111/1471-0528.15022

28. Farrell RM, Agatisa PK, Nutter B. What women want: lead considerations for current and future applications of noninvasive prenatal testing in prenatal care. *Birth*. 2014;41(3):276-282. doi:10.1111/birt.12113

29. Munro S, Sou J, Zhang W, et al. Attitudes toward prenatal screening for chromosomal abnormalities: A focus group study. *Women and Birth*. September 2018:S1871519218303597. doi:10.1016/j.wombi.2018.09.006

30. Ngan OMY, Yi H, Wong SYS, Sahota D, Ahmed S. Obstetric professionals’ perceptions of non-invasive prenatal testing for Down syndrome: clinical usefulness compared with existing tests and ethical implications. *BMC Pregnancy Childbirth*. 2017;17(1):285. doi:10.1186/s12884-017-1474-6

**Table 1: Search strategy based on the PICO approach**

| **Concept** | | **Search terms** | **Combination of concepts** |
| --- | --- | --- | --- |
| #1 Population | #1.1 Prenatal tests | prenatal care, prenatal cares, prenatal diagnosis, prenatal diagnose, prenatal screening, prenatal screenings, antenatal diagnose, antenatal diagnosis, antenatal screening, antenatal screenings, intrauterine diagnose, intrauterine diagnosis, prenatal diagnosis [Mesh], prenatal care [Mesh] | (#1.1 AND #1.2)  AND #2 AND  (#3.1 OR 3.2) |
|  | #1.2 Fetal conditions | Fetal, fetus, foetus, fetal structure, fetal structures, mummified fetus, retained fetus, fetal tissue, fetal tissues, fetal disease, fetal diseases, embryopathy, embryopathies, fetus [Mesh], fetal disease [Mesh] |  |
| #2 Intervention/  Measurements | DCEs | discrete choice experiment, discrete choice experiments, discrete choice model, discrete choice models, discrete choice modelings, discrete choice conjoint experiment, path-worth utilities, functional measurement, paired comparison, pairwise choices, conjoint analysis, conjoint measurement, conjoint study, conjoint studies, conjoint choice experiment, conjoint choice experiments, stated preference |  |
| #3 Outcomes | #3.1 Attributes | attribute, attributes, component, components, dimension, dimensions, item, items |  |
|  | #3.2 Valuation | monetary value, money value, monetary measurement, monetary measurements willingness-to-pay, benefit, benefits |  |

**Table 2: Description of the DCE studies included in this review**

| **Authors, Country** | **Year** | **Prenatal test** | **Attribute (number of attribute levels)** | | | | | | |
| --- | --- | --- | --- | --- | --- | --- | --- | --- | --- |
|  |  |  | Level of information/ Genetic information | Time in pregnancy when results received | Time to result interval | Time in pregnancy when test is conducted | Accuracy/ Detection rate | Risk of miscarriage/ Safety | Cost |
| Chan et al. (Hongkong, China) | 2009 | Conventional karyotyping test or rapid diagnostic tests | 2 |  | 4 |  |  |  | 4 |
| Barrett et al. (Singapore) | 2017 | NIPT and invasive for DS | 2 | 3 |  |  | 3 | 2 |  |
| *Lynn et al. (UK) | 2015 | Third trimester ultrasound scan |  |  |  |  | 2 |  | 4 |
| Hill et al. (UK) | 2017 | NIPD for sickle-cell |  | 4 |  |  | 4 | 2 |  |
| Lund et al. (Denmark) | 2018 | NIPT and invasive for DS | 2 | 3 |  |  | 3 | 2 |  |
| Hill et al. (UK) | 2014 | NIPD on cystic fibrosis |  | 4 |  |  | 4 | 2 |  |
| Hill et al. (multi-country) | 2016 | NIPT and invasive for DS | 2 | 3 |  |  | 3 | 2 |  |
| Ryan et al. (UK) | 2005 | Rapid diagnosis for DS | 2 |  | 4 |  |  |  | 4 |
| Bishop et al. (UK) | 2004 | Screening test for DS |  |  |  | 4 | 4 | 4 |  |
| Carroll et al. (UK) | 2013 | Screening test for DS |  |  | 2 | 2 | 2 |  | 4 |
| Lewis et al. (Australia) | 2006 | Screening test for DS |  |  |  | 4 | 4 | 4 |  |
| **Beulen et al. (Netherlands) | 2015 | Prenatal screening and diagnostic tests | 3 |  | 3 | 3 | 4 | 3 | 4 |
| Hill et al. (UK) |  |  | 2 | 3 |  |  | 3 | 2 | 2 |

**Notes**: * two additional attributes: ‘*heal-care professional*’, ‘*non-medical information*’; ** one additional attribute: ‘*false positive rate*’

**Abbreviations:** NIPT = non-invasive prenatal test; DS = Down syndrome

**Figure 1: PRISMA-P flow diagram for searching article for DCE in prenatal screening**

No. of articles excluded (n= 45):

- Not DCE methodology

- Full-text is not available in English

- Not prenatal screening tests-related

Literature search

PubMed: 27

Embase: 13

Cochrane library: 0

Econlit: 0

Web of science: 10

ProQuest Dissertations and Theses Global: 20

Remove duplicates, non-English publications

(n= 15)

No. of articles screened on basis of titles and abstracts

(n=55)

No. of articles from reference list of the retrieved studies

(n=4)

No. of full text articles accessed for eligibility

(n=10)

Full-text articles excluded due to duplication

(n= 1)

No. of articles included for final data extraction

(n=13)

**Identification**

**Screening**

**Eligibility**

**Included**
