# Supplementary material for: Development of a discrete choice experiment questionnaire to elicit preferences by pregnant women and policymakers for the expansion of non-invasive prenatal screening
Source: PLoS One. 2023 Jun 23;18(6):e0287653. doi: 10.1371/journal.pone.0287653 (PMC10289448; doi:10.1371/journal.pone.0287653)
Supplement: S2 Text — (DOCX) [file pone.0287653.s002.docx]

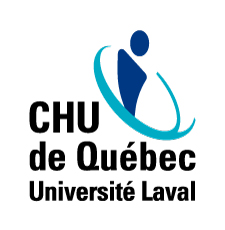

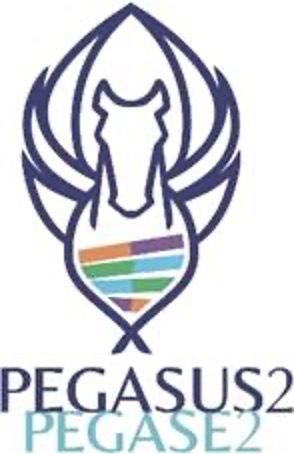


**Project: *Women's and policymakers’ valuation of the expansion of a NIPS-based prenatal screening program: A Discrete Choice Experiment approach study***

**Interview Guide**

The interview topics are intended to help us build an instrument to measure preferences for a new test to detect a possible health problem in a future child (in prenatal screening), based on concerns shared by pregnant women and decision makers.

We will do a first round with a general question, then a second round with more specific questions. Please feel free to tell us anything you would like to say. You can of course react to something someone else has said.

**General Question**

- According to you, what would justify offering a test to a pregnant woman to detect a possible genetic problem in her future baby?

**Specific Questions**

- To what extent should the costs borne by a pregnant woman and her family to receive a prenatal test influence the decision on whether to offer the test in the public system?
- How important do you think is the uncertainty about the expected outcome of a test (its performance or the rate of false positives and false negatives) in deciding whether to offer the test in the public system?
- How important do you think is the degree of physical disability associated with a chromosomal abnormality in deciding whether to offer the test in the public system?
- How important do you think is the uncertainty about the degree of physical disability that results from an abnormality detected by the test in the decision whether to offer the test in the public system?
- How important do you think is the degree of intellectual disability associated with a chromosomal abnormality in deciding whether to offer the test in the public system?
- How important do you think the uncertainty about the degree of intellectual disability that results from an abnormality detected by the test is in the decision whether to offer the test in the public system?
- What other considerations should influence the decision about whether to offer the test in the public system?
