# Supplementary material for: Development of a discrete choice experiment questionnaire to elicit preferences by pregnant women and policymakers for the expansion of non-invasive prenatal screening
Source: PLoS One. 2023 Jun 23;18(6):e0287653. doi: 10.1371/journal.pone.0287653 (PMC10289448; doi:10.1371/journal.pone.0287653)
Supplement: S3 Text — (DOCX) [file pone.0287653.s003.docx]

**Supporting information file 3: DCE pilot study design**

PILOT STUDY of 33 subjects with 8 tasks: 7 comparison tasks each involving 2 options + 1 choice task with a dominant option

- 8 attributes (number of levels) in the project

A(2), B(2), C(2), D(2), E(2), F(3), H(3)

- Choice of 22 options to compare two by two from a d-optimal design:

Options A B C D E F G H

1 2 2 2 2 1 3 2 2

2 2 2 2 1 2 3 1 3

3 2 2 2 1 1 2 3 1

4 2 2 1 2 2 1 1 2

5 2 2 1 2 1 1 2 3

6 2 1 2 2 2 2 1 1

7 2 1 2 1 2 1 3 2

8 2 1 2 1 1 3 1 1

9 2 1 1 2 1 3 1 3

10 2 1 1 2 1 2 2 1

11 2 1 1 1 2 3 2 2

12 1 2 2 2 1 1 3 2

13 1 2 2 1 2 2 3 3

14 1 2 2 1 2 1 1 1

15 1 2 1 2 2 2 3 1

16 1 2 1 1 1 2 2 2

17 1 1 2 2 2 2 2 2

18 1 1 2 1 2 1 2 3

19 1 1 2 1 1 2 1 3

20 1 1 1 2 2 3 1 1

21 1 1 1 1 2 3 3 3

22 1 1 1 1 1 1 1 2

These 22 options compared 2 by 2 yield 231 pairs.

We will therefore need n=33 subjects in the pilot study, with 7 comparison tasks for each (33*7=231).

- One dominant choice task for each subject

option1 option2

2-1-2-1-1-1-1-1 1-2-1-2-2-2-2-3

- List of 7 tasks to be compared by subject:

------------------------------------ sujet=1 -------------------------------------

option1 option2

1-2-2-2-1-1-3-2 1-1-2-2-2-2-2-2

2-1-1-2-1-2-2-1 1-1-2-2-2-2-2-2

2-2-1-2-2-1-1-2 1-2-1-2-2-2-3-1

2-2-1-2-2-1-1-2 2-1-2-2-2-2-1-1

2-2-2-2-1-3-2-2 2-1-2-2-2-2-1-1

2-2-1-2-1-1-2-3 2-1-2-2-2-2-1-1

1-1-2-1-1-2-1-3 1-1-1-1-1-1-1-2

------------------------------------ sujet=2 -------------------------------------

option1 option2

2-1-2-1-1-3-1-1 1-2-2-1-2-2-3-3

2-2-2-2-1-3-2-2 2-1-2-1-1-3-1-1

2-1-2-1-2-1-3-2 1-2-2-2-1-1-3-2

1-1-2-1-2-1-2-3 1-1-2-1-1-2-1-3

1-1-1-2-2-3-1-1 1-1-1-1-1-1-1-2

2-1-1-1-2-3-2-2 1-1-1-1-1-1-1-2

2-2-2-2-1-3-2-2 1-1-2-2-2-2-2-2

------------------------------------ sujet=3 -------------------------------------

option1 option2

2-2-2-1-2-3-1-3 2-1-1-1-2-3-2-2

2-2-1-2-2-1-1-2 1-2-1-1-1-2-2-2

2-1-1-1-2-3-2-2 1-1-1-1-2-3-3-3

1-2-1-2-2-2-3-1 1-1-2-1-2-1-2-3

2-2-2-1-1-2-3-1 2-2-1-2-2-1-1-2

2-2-1-2-1-1-2-3 1-1-2-1-1-2-1-3

2-2-2-2-1-3-2-2 2-1-1-2-1-2-2-1

------------------------------------ sujet=4 -------------------------------------

option1 option2

2-1-2-2-2-2-1-1 1-1-2-1-2-1-2-3

2-2-1-2-2-1-1-2 2-1-2-1-1-3-1-1

1-1-2-2-2-2-2-2 1-1-1-1-2-3-3-3

1-2-2-2-1-1-3-2 1-2-1-2-2-2-3-1

2-1-1-2-1-3-1-3 1-2-2-1-2-1-1-1

2-2-2-1-1-2-3-1 1-1-2-1-2-1-2-3

2-1-1-2-1-2-2-1 1-1-1-1-1-1-1-2

------------------------------------ sujet=5 -------------------------------------

option1 option2

2-1-1-2-1-3-1-3 1-2-1-2-2-2-3-1

2-1-1-2-1-3-1-3 1-1-2-1-2-1-2-3

2-2-2-1-1-2-3-1 1-2-2-1-2-1-1-1

2-1-2-2-2-2-1-1 2-1-1-2-1-3-1-3

2-2-1-2-2-1-1-2 2-2-1-2-1-1-2-3

1-2-2-1-2-1-1-1 1-2-1-1-1-2-2-2

1-2-2-2-1-1-3-2 1-1-1-2-2-3-1-1

------------------------------------ sujet=6 -------------------------------------

option1 option2

2-1-1-2-1-2-2-1 1-2-1-2-2-2-3-1

2-2-2-2-1-3-2-2 1-2-2-1-2-2-3-3

1-1-2-1-2-1-2-3 1-1-1-1-2-3-3-3

2-1-1-1-2-3-2-2 1-2-2-1-2-2-3-3

2-2-2-2-1-3-2-2 2-2-2-1-2-3-1-3

1-2-2-2-1-1-3-2 1-1-1-1-2-3-3-3

2-2-2-2-1-3-2-2 1-2-2-2-1-1-3-2

------------------------------------ sujet=7 -------------------------------------

option1 option2

2-1-1-2-1-2-2-1 2-1-1-1-2-3-2-2

1-1-2-1-2-1-2-3 1-1-1-1-1-1-1-2

2-1-1-2-1-3-1-3 2-1-1-2-1-2-2-1

2-2-1-2-1-1-2-3 1-2-2-1-2-1-1-1

2-2-2-1-1-2-3-1 1-2-2-2-1-1-3-2

2-2-1-2-1-1-2-3 2-1-1-1-2-3-2-2

2-2-1-2-2-1-1-2 1-2-2-1-2-2-3-3

------------------------------------ sujet=8 -------------------------------------

option1 option2

1-2-1-1-1-2-2-2 1-1-2-1-1-2-1-3

1-1-2-1-1-2-1-3 1-1-1-2-2-3-1-1

2-2-1-2-2-1-1-2 2-1-1-2-1-2-2-1

2-2-2-2-1-3-2-2 1-1-1-2-2-3-1-1

2-2-2-1-2-3-1-3 2-2-1-2-2-1-1-2

2-2-2-1-1-2-3-1 2-1-1-2-1-3-1-3

2-1-2-1-2-1-3-2 2-1-1-2-1-3-1-3

------------------------------------ sujet=9 -------------------------------------

option1 option2

1-2-2-2-1-1-3-2 1-2-2-1-2-1-1-1

2-1-2-1-1-3-1-1 1-1-2-1-2-1-2-3

2-2-1-2-2-1-1-2 1-2-2-2-1-1-3-2

2-2-2-1-1-2-3-1 1-1-2-2-2-2-2-2

2-2-2-2-1-3-2-2 2-1-1-2-1-3-1-3

2-1-1-2-1-3-1-3 1-1-1-1-1-1-1-2

2-2-1-2-2-1-1-2 1-1-1-1-1-1-1-2

------------------------------------ sujet=10 ------------------------------------

option1 option2

2-2-2-2-1-3-2-2 2-1-2-1-2-1-3-2

1-2-2-1-2-2-3-3 1-1-1-1-2-3-3-3

2-2-2-1-1-2-3-1 1-1-1-2-2-3-1-1

2-2-2-1-2-3-1-3 2-1-1-2-1-2-2-1

2-2-1-2-2-1-1-2 1-1-2-1-1-2-1-3

2-2-2-2-1-3-2-2 2-2-1-2-2-1-1-2

1-2-2-2-1-1-3-2 1-2-2-1-2-2-3-3

------------------------------------ sujet=11 ------------------------------------

option1 option2

1-2-2-1-2-1-1-1 1-1-2-1-2-1-2-3

2-2-2-1-2-3-1-3 1-1-2-1-2-1-2-3

2-1-2-2-2-2-1-1 1-1-2-2-2-2-2-2

2-1-2-2-2-2-1-1 1-2-2-2-1-1-3-2

2-2-1-2-1-1-2-3 1-1-2-2-2-2-2-2

1-2-1-1-1-2-2-2 1-1-1-1-2-3-3-3

2-2-1-2-2-1-1-2 2-1-1-1-2-3-2-2

------------------------------------ sujet=12 ------------------------------------

option1 option2

2-2-2-2-1-3-2-2 1-1-1-1-1-1-1-2

1-2-2-1-2-2-3-3 1-1-2-2-2-2-2-2

2-2-2-2-1-3-2-2 1-2-1-2-2-2-3-1

2-2-2-1-1-2-3-1 1-1-2-1-1-2-1-3

1-2-2-1-2-2-3-3 1-1-2-1-1-2-1-3

2-1-2-1-1-3-1-1 1-2-2-2-1-1-3-2

2-1-1-2-1-3-1-3 1-1-2-1-1-2-1-3

------------------------------------ sujet=13 ------------------------------------

option1 option2

2-1-2-1-2-1-3-2 1-1-2-1-1-2-1-3

2-2-1-2-1-1-2-3 1-2-1-2-2-2-3-1

2-1-1-2-1-3-1-3 1-1-1-2-2-3-1-1

2-1-1-2-1-2-2-1 1-2-2-2-1-1-3-2

2-2-2-2-1-3-2-2 1-1-2-1-2-1-2-3

1-2-2-1-2-2-3-3 1-1-2-1-2-1-2-3

2-1-2-1-1-3-1-1 2-1-1-2-1-2-2-1

------------------------------------ sujet=14 ------------------------------------

option1 option2

2-2-1-2-1-1-2-3 1-1-1-2-2-3-1-1

2-1-2-1-1-3-1-1 1-2-2-1-2-1-1-1

2-2-1-2-1-1-2-3 1-2-1-1-1-2-2-2

2-2-2-1-2-3-1-3 1-2-1-2-2-2-3-1

2-1-2-1-2-1-3-2 1-2-1-2-2-2-3-1

2-1-1-2-1-2-2-1 1-2-1-1-1-2-2-2

2-1-1-2-1-2-2-1 1-2-2-1-2-1-1-1

------------------------------------ sujet=15 ------------------------------------

option1 option2

2-1-2-2-2-2-1-1 2-1-1-2-1-2-2-1

1-2-1-2-2-2-3-1 1-1-1-1-2-3-3-3

2-2-1-2-2-1-1-2 2-1-1-2-1-3-1-3

1-1-2-2-2-2-2-2 1-1-1-1-1-1-1-2

2-2-2-1-1-2-3-1 1-1-1-1-1-1-1-2

2-1-1-2-1-2-2-1 1-1-1-2-2-3-1-1

2-1-1-2-1-3-1-3 1-1-2-2-2-2-2-2

------------------------------------ sujet=16 ------------------------------------

option1 option2

2-2-2-1-2-3-1-3 1-1-1-2-2-3-1-1

2-1-2-1-1-3-1-1 1-2-1-2-2-2-3-1

1-2-2-1-2-1-1-1 1-1-1-1-1-1-1-2

2-1-2-2-2-2-1-1 2-1-1-1-2-3-2-2

2-2-2-2-1-3-2-2 1-1-1-1-2-3-3-3

1-2-2-1-2-2-3-3 1-2-1-1-1-2-2-2

2-2-1-2-1-1-2-3 1-2-2-2-1-1-3-2

------------------------------------ sujet=17 ------------------------------------

option1 option2

2-2-2-1-1-2-3-1 1-1-1-1-2-3-3-3

2-2-2-1-2-3-1-3 1-1-2-2-2-2-2-2

1-1-1-2-2-3-1-1 1-1-1-1-2-3-3-3

2-1-2-2-2-2-1-1 1-1-2-1-1-2-1-3

1-1-2-2-2-2-2-2 1-1-1-2-2-3-1-1

2-2-2-2-1-3-2-2 1-2-1-1-1-2-2-2

2-1-1-2-1-2-2-1 1-1-2-1-2-1-2-3

------------------------------------ sujet=18 ------------------------------------

option1 option2

1-2-1-2-2-2-3-1 1-1-1-2-2-3-1-1

2-1-2-1-2-1-3-2 1-1-2-1-2-1-2-3

2-1-1-1-2-3-2-2 1-2-1-2-2-2-3-1

2-2-1-2-2-1-1-2 1-2-2-1-2-1-1-1

2-1-2-1-2-1-3-2 2-1-2-1-1-3-1-1

1-2-2-2-1-1-3-2 1-2-1-1-1-2-2-2

2-1-1-2-1-3-1-3 2-1-1-1-2-3-2-2

------------------------------------ sujet=19 ------------------------------------

option1 option2

1-2-2-2-1-1-3-2 1-1-2-1-1-2-1-3

2-1-1-1-2-3-2-2 1-1-2-1-2-1-2-3

2-2-2-2-1-3-2-2 1-1-2-1-1-2-1-3

2-2-2-1-2-3-1-3 1-1-2-1-1-2-1-3

2-2-1-2-2-1-1-2 1-1-1-2-2-3-1-1

2-1-2-2-2-2-1-1 1-1-1-2-2-3-1-1

2-2-1-2-1-1-2-3 2-1-1-2-1-2-2-1

------------------------------------ sujet=20 ------------------------------------

option1 option2

2-1-2-1-1-3-1-1 1-2-1-1-1-2-2-2

1-2-2-1-2-1-1-1 1-1-1-1-2-3-3-3

2-2-1-2-1-1-2-3 1-1-2-1-2-1-2-3

2-2-2-1-1-2-3-1 2-1-1-2-1-2-2-1

2-1-1-1-2-3-2-2 1-1-2-2-2-2-2-2

2-2-2-1-2-3-1-3 2-1-2-2-2-2-1-1

1-2-2-1-2-1-1-1 1-1-2-1-1-2-1-3

------------------------------------ sujet=21 ------------------------------------

option1 option2

2-1-2-1-2-1-3-2 1-2-2-1-2-1-1-1

2-1-2-1-2-1-3-2 1-1-2-2-2-2-2-2

2-2-2-1-2-3-1-3 2-1-2-1-1-3-1-1

2-2-1-2-1-1-2-3 1-1-1-1-2-3-3-3

2-2-2-1-1-2-3-1 2-1-2-1-1-3-1-1

2-2-2-1-1-2-3-1 1-2-1-2-2-2-3-1

2-2-2-1-2-3-1-3 2-1-2-1-2-1-3-2

------------------------------------ sujet=22 ------------------------------------

option1 option2

2-1-2-1-2-1-3-2 1-2-2-1-2-2-3-3

2-2-1-2-1-1-2-3 2-1-2-1-2-1-3-2

2-2-2-1-1-2-3-1 1-2-1-1-1-2-2-2

2-2-2-1-2-3-1-3 2-2-2-1-1-2-3-1

1-1-2-2-2-2-2-2 1-1-2-1-1-2-1-3

2-2-2-1-2-3-1-3 1-1-1-1-1-1-1-2

2-2-1-2-2-1-1-2 1-1-2-2-2-2-2-2

------------------------------------ sujet=23 ------------------------------------

option1 option2

2-2-1-2-1-1-2-3 1-2-2-1-2-2-3-3

2-1-1-2-1-3-1-3 1-1-1-1-2-3-3-3

2-1-1-1-2-3-2-2 1-2-2-2-1-1-3-2

2-2-2-1-2-3-1-3 1-2-2-2-1-1-3-2

2-1-2-1-1-3-1-1 1-1-1-2-2-3-1-1

2-2-2-1-2-3-1-3 2-2-1-2-1-1-2-3

1-1-1-1-2-3-3-3 1-1-1-1-1-1-1-2

------------------------------------ sujet=24 ------------------------------------

option1 option2

1-2-2-1-2-1-1-1 1-1-1-2-2-3-1-1

2-1-2-2-2-2-1-1 1-1-1-1-2-3-3-3

2-2-1-2-2-1-1-2 2-1-2-1-2-1-3-2

2-2-1-2-1-1-2-3 2-1-2-1-1-3-1-1

1-2-1-1-1-2-2-2 1-1-1-2-2-3-1-1

2-1-1-1-2-3-2-2 1-2-2-1-2-1-1-1

2-2-1-2-1-1-2-3 2-1-1-2-1-3-1-3

------------------------------------ sujet=25 ------------------------------------

option1 option2

2-1-2-1-1-3-1-1 2-1-1-2-1-3-1-3

1-2-2-1-2-2-3-3 1-1-1-1-1-1-1-2

2-2-2-1-2-3-1-3 1-2-2-1-2-2-3-3

2-2-2-1-1-2-3-1 2-2-1-2-1-1-2-3

2-2-2-1-1-2-3-1 2-1-1-1-2-3-2-2

2-1-2-1-1-3-1-1 1-1-2-1-1-2-1-3

2-1-2-1-2-1-3-2 1-1-1-1-1-1-1-2

------------------------------------ sujet=26 ------------------------------------

option1 option2

1-2-2-1-2-2-3-3 1-2-1-2-2-2-3-1

2-2-2-2-1-3-2-2 2-1-1-1-2-3-2-2

2-1-2-1-2-1-3-2 1-1-1-1-2-3-3-3

2-1-2-1-2-1-3-2 1-1-1-2-2-3-1-1

2-2-2-2-1-3-2-2 2-2-1-2-1-1-2-3

2-2-2-1-1-2-3-1 2-1-2-1-2-1-3-2

2-1-2-2-2-2-1-1 1-2-1-1-1-2-2-2

------------------------------------ sujet=27 ------------------------------------

option1 option2

2-1-2-1-1-3-1-1 1-1-2-2-2-2-2-2

1-2-2-1-2-1-1-1 1-2-1-2-2-2-3-1

2-2-2-1-2-3-1-3 2-1-1-2-1-3-1-3

1-2-1-1-1-2-2-2 1-1-1-1-1-1-1-2

2-1-1-2-1-2-2-1 1-2-2-1-2-2-3-3

1-2-1-1-1-2-2-2 1-1-2-1-2-1-2-3

2-2-2-2-1-3-2-2 1-2-2-1-2-1-1-1

------------------------------------ sujet=28 ------------------------------------

option1 option2

1-1-2-1-1-2-1-3 1-1-1-1-2-3-3-3

2-2-2-2-1-3-2-2 2-2-2-1-1-2-3-1

2-2-1-2-2-1-1-2 1-1-1-1-2-3-3-3

2-1-1-2-1-3-1-3 1-2-2-1-2-2-3-3

2-1-2-1-1-3-1-1 1-1-1-1-1-1-1-2

1-2-1-2-2-2-3-1 1-1-1-1-1-1-1-2

2-1-2-1-2-1-3-2 2-1-1-2-1-2-2-1

------------------------------------ sujet=29 ------------------------------------

option1 option2

2-2-2-1-2-3-1-3 1-2-2-1-2-1-1-1

2-1-1-2-1-2-2-1 1-1-2-1-1-2-1-3

2-1-1-2-1-2-2-1 1-1-1-1-2-3-3-3

2-2-2-1-2-3-1-3 1-1-1-1-2-3-3-3

2-2-1-2-2-1-1-2 1-1-2-1-2-1-2-3

2-2-1-2-1-1-2-3 1-1-1-1-1-1-1-2

2-1-2-2-2-2-1-1 1-1-1-1-1-1-1-2

------------------------------------ sujet=30 ------------------------------------

option1 option2

1-2-2-1-2-2-3-3 1-1-1-2-2-3-1-1

2-1-1-2-1-3-1-3 1-2-1-1-1-2-2-2

2-1-1-1-2-3-2-2 1-1-2-1-1-2-1-3

2-1-2-2-2-2-1-1 1-2-2-1-2-2-3-3

1-2-2-1-2-2-3-3 1-2-2-1-2-1-1-1

2-1-2-1-2-1-3-2 2-1-1-1-2-3-2-2

2-2-2-1-2-3-1-3 1-2-1-1-1-2-2-2

------------------------------------ sujet=31 ------------------------------------

option1 option2

2-1-2-1-2-1-3-2 1-2-1-1-1-2-2-2

1-1-2-2-2-2-2-2 1-1-2-1-2-1-2-3

2-1-2-2-2-2-1-1 1-2-1-2-2-2-3-1

2-1-2-1-1-3-1-1 1-1-1-1-2-3-3-3

1-1-2-1-2-1-2-3 1-1-1-2-2-3-1-1

2-1-1-1-2-3-2-2 1-1-1-2-2-3-1-1

2-2-2-1-1-2-3-1 2-1-2-2-2-2-1-1

------------------------------------ sujet=32 ------------------------------------

option1 option2

1-2-1-2-2-2-3-1 1-1-2-1-1-2-1-3

1-2-2-1-2-1-1-1 1-1-2-2-2-2-2-2

2-1-2-2-2-2-1-1 1-2-2-1-2-1-1-1

2-1-2-2-2-2-1-1 2-1-2-1-1-3-1-1

1-2-2-2-1-1-3-2 1-1-2-1-2-1-2-3

2-1-1-2-1-3-1-3 1-2-2-2-1-1-3-2

1-2-1-1-1-2-2-2 1-1-2-2-2-2-2-2

------------------------------------ sujet=33 ------------------------------------

option1 option2

1-2-2-2-1-1-3-2 1-1-1-1-1-1-1-2

2-1-2-2-2-2-1-1 2-1-2-1-2-1-3-2

1-2-1-2-2-2-3-1 1-1-2-2-2-2-2-2

2-1-2-1-1-3-1-1 2-1-1-1-2-3-2-2

1-2-1-2-2-2-3-1 1-2-1-1-1-2-2-2

2-2-2-1-1-2-3-1 1-2-2-1-2-2-3-3

2-1-1-1-2-3-2-2 1-2-1-1-1-2-2-2
