# Supplementary material for: Development of a discrete choice experiment questionnaire to elicit preferences by pregnant women and policymakers for the expansion of non-invasive prenatal screening
Source: PLoS One. 2023 Jun 23;18(6):e0287653. doi: 10.1371/journal.pone.0287653 (PMC10289448; doi:10.1371/journal.pone.0287653)
Supplement: Attachment — Submitted filename: Response to Reviewers.docx [file pone.0287653.s004.docx]

AUTHORS’ RESPONSE

| **No.** | **Journal requirements** | **Authors’ responses** |
| --- | --- | --- |
| 1 | Please ensure that your manuscript meets PLOS ONE's style requirements, including those for file naming. The PLOS ONE style templates can be found at  <https://journals.plos.org/plosone/s/file?id=wjVg/PLOSOne_formatting_sample_main_body.pdf> and  <https://journals.plos.org/plosone/s/file?id=ba62/PLOSOne_formatting_sample_title_authors_affiliations.pdf> | This article was reformatted to meet the journal’s style requirement. |
| 2 | Please provide additional details regarding participant consent. In the ethics statement in the Methods and online submission information, please ensure that you have specified what type you obtained (for instance, written or verbal, and if verbal, how it was documented and witnessed). If your study included minors, state whether you obtained consent from parents or guardians. If the need for consent was waived by the ethics committee, please include this information. | Additional details were provided in the text:  “Written informed consent was obtained from all participants. Pregnant women signed consent form after being informed of the study by the two researchers (HMN and CL). Policymakers provided their electronic signatures on the consent form, which was returned to the researchers via email.” (page 5, line 82)  We did not include minors in our study. Only pregnant women who aged 18 years or above were included in our study. This information was stated in the methodology section:  “The inclusion criteria for pregnant women were primigravida, aged 18 years or older, and consulting the obstetric department of the CHUL in Québec City, Canada, for their first prenatal echography.” (page 7, line 121) |
| 3 | We note that the grant information you provided in the ‘Funding Information’ and ‘Financial Disclosure’ sections do not match.  When you resubmit, please ensure that you provide the correct grant numbers for the awards you received for your study in the ‘Funding Information’ section. | The same information was checked and provided in both “Funding information” and “Financial Disclosure” sections in the revised submission. |
| 4 | Thank you for stating the following financial disclosure:  “Financial support for this study was provided entirely by a grant from the PEGASUS2 project funded by Génome Canada (with Génome Québec; Génome BC; Génome Alberta; the Québec Ministère de l’Enseignement supérieur, de la recherche, de la science et de la technologie; the Fonds de recherche Québec – Santé; la Fondation de l’Université Laval; and the Centre de recherche du CHU de Québec) – Grant number: LSARP2012-4523, and the Canadian Institutes for Health Research - Grant number: GPH129342. The funders had no role in study design, data collection and analysis, decision to publish, or preparation of the manuscript. Jason R Guertin is a FRQS Research Scholar Junior 1 (Award #266460).”  Please state what role the funders took in the study.  If the funders had no role, please state: ""The funders had no role in study design, data collection and analysis, decision to publish, or preparation of the manuscript.""  If this statement is not correct you must amend it as needed.  Please include this amended Role of Funder statement in your cover letter; we will change the online submission form on your behalf. | We made the changes, as suggested by the editor. The sentence was also added to the “Financial disclosure” section in the cover letter as follows:  “Financial support for this study was provided entirely by a grant from the PEGASUS2 project funded by Génome Canada (with Génome Québec; Génome BC; Génome Alberta; the Québec Ministère de l’Enseignement supérieur, de la recherche, de la science et de la technologie; the Fonds de recherche Québec – Santé; la Fondation de l’Université Laval; and the Centre de recherche du CHU de Québec) – Grant number: LSARP2012-4523, and the Canadian Institutes for Health Research - Grant number: GPH129342. The funders had no role in study design, data collection and analysis, decision to publish, or preparation of the manuscript. Jason R Guertin is a FRQS Research Scholar Junior 1 (Award #266460).” |
| 5 | Comments to the Author |  |
|  | 1. Is the manuscript technically sound, and do the data support the conclusions?  The manuscript must describe a technically sound piece of scientific research with data that supports the conclusions. Experiments must have been conducted rigorously, with appropriate controls, replication, and sample sizes. The conclusions must be drawn appropriately based on the data presented.  Reviewer #1: Partly  Reviewer #2: Partly | The manuscript was revised and rewritten taking into account all reviewers’ comments to, hopefully, improve the presentation of its scientific quality. |
|  | 2. Has the statistical analysis been performed appropriately and rigorously?  Reviewer #1: I Don't Know  Reviewer #2: Yes | The analyses performed following statistical methods guidelines for discrete choice experiments. A statistician of the department of mathematics and statistics at Université Laval has been consulted for the statistical analysis plan. |
|  | 3. Have the authors made all data underlying the findings in their manuscript fully available?  The [PLOS Data policy](http://www.plosone.org/static/policies.action#sharing) requires authors to make all data underlying the findings described in their manuscript fully available without restriction, with rare exception (please refer to the Data Availability Statement in the manuscript PDF file). The data should be provided as part of the manuscript or its supporting information, or deposited to a public repository. For example, in addition to summary statistics, the data points behind means, medians and variance measures should be available. If there are restrictions on publicly sharing data—e.g. participant privacy or use of data from a third party—those must be specified.  Reviewer #1: Yes  Reviewer #2: Yes |  |
|  | 4. Is the manuscript presented in an intelligible fashion and written in standard English?  PLOS ONE does not copyedit accepted manuscripts, so the language in submitted articles must be clear, correct, and unambiguous. Any typographical or grammatical errors should be corrected at revision, so please note any specific errors here.  Reviewer #1: Yes  Reviewer #2: Yes |  |
|  | 5. Review Comments to the Author  Please use the space provided to explain your answers to the questions above. You may also include additional comments for the author, including concerns about dual publication, research ethics, or publication ethics. (Please upload your review as an attachment if it exceeds 20,000 characters) | Please, see our replies above opposite to the different Journal’s requirement items, when relevant. |
|  | **Reviewer #1** |  |
|  | 1) ABSTRACT: a) Conclusion in the Abstract sounds more like the "outcome" or "benefit" of the study rather than the conclusion. The conclusion may be rephrased to reflect the Results of the study. b) A very brief on the methdology of Qualitative study and the pilot study may be helpful | a) The conclusion of the abstract was rewritten as follows:  “Conclusion. It is possible to reach a consensus on the construction of a DCE instrument intended to be administered to pregnant women and policymakers. However, complete validation of the consensual instrument is limited because there are too few policymakers to statistically ascertain the relevance of the attributes and their levels.” (page 2, line 18)  b) Additional information on qualitative study and the pilot project was added to the methodology of the abstract as follows:  “Methods: A DCE development study was conducted. The methods employed included a literature review, a qualitative study (based on individual semi-structured interviews, consultations, and a focus group discussion) with pregnant women and policymakers, and a pilot project with 33 pregnant women to validate the first version of the instrument and test the feasibility of its administration.” (page 2, line 7) |
|  | 2) LINE 56 - MESH terms | Thank you for pointing the mistake out. The word has been rewritten in capital letters in the revised manuscript as follows:  “MESH terms” (page 6, line 93) |
|  | 3) Less than 50% of the 68 women who were interested in the pilot study completed the survey. May discuss the possible reason in the Discussion section | The low response rate was considered as one of the study limitations. The following paragraph was added in the discussion section as follows:  “This study has some limitations. First, about half the individuals who agreed to participate (51%) did not complete the survey. Of these, 78% quit the survey after reading the informed consent form, whereas the rest (22%) stopped answering the first question. The reasons why many potential participants refused to take part in the study were not investigated. However, this observation is commonly reported in DCE studies. The main reasons for the low participation rate presented in the literature are the complexity of the task asked, sensitivity of the study topic, cognitive burden of the DCE questionnaire, and having poor familiarity with trade-off tasks [39, 40].” (page 22, line 348) |
|  | 4) CONCUSIONS: do not reflect the results, may need re-phrasing | We rephrased the conclusion as follows:  “Having a common DCE instrument to be administered to the beneficiaries of an intervention and policymakers is expected to allow the identification of similarities and differences in the value experiences of both groups. These quantitative data should help policymakers evaluate whether the beneficiaries’ perspective that they are mandated to consider is sufficiently taken into account in their recommendations on the provision of an intervention for the population.  This study showed that it is possible to obtain a consensus list of attributes from pregnant women and policymakers regarding the expansion of NIPS-based programs. Therefore, a single DCE instrument can be constructed to compare preferences between the demand and supply sides of healthcare systems for the eventual addition of new conditions to be screened in the program. However, complete validation of the instrument is limited because there may be too few policymakers to statistically ascertain the relevance of the attributes and their levels.” (page 24, line 375) |
|  | 5) Limitations of the study might be prased clearly | Study limitations section was added to the discussion as follows:  “This study has some limitations. First, about half the individuals who agreed to participate (51%) did not complete the survey. Of these, 78% quit the survey after reading the informed consent form, whereas the rest (22%) stopped answering the first question. The reasons why many potential participants refused to take part in the study were not investigated. However, this observation is commonly reported in DCE studies. The main reasons for the low participation rate presented in the literature are the complexity of the task asked, sensitivity of the study topic, cognitive burden of the DCE questionnaire, and having poor familiarity with trade-off tasks [39, 40].  Second, we were unable to test the instrument with policymakers in the pilot project because of the limited pool of individuals who voted as members of health policy deliberating committees. Indeed, the recruitment of policymakers must consider the fact that their participation would be required not only in the development phase of DCE instrument construction but also in the administration phase of the final version of the DCE questionnaire, which requires a larger sample size. The main consequence of this limitation is that some attributes retained in the final version of the DCE instrument may not be considered essential by policymakers; hence, they may not be statistically significant when administered in a full-scale study.  Third, only primigravidae were included in the interviews. The inclusion of a homogenous population of gravidae in the instrument construction was considered to limit the complexity of the experimental design. We cannot exclude the possibility that subgroups of pregnant women might value instrument attributes differently.  Finally, for practical reasons, only pregnant women between 28-30 weeks’ gestation were included in the testing phase of the study. In addition, these women can be whether or not primigravidae, as required by qualitative studies. This may have affected the results. However, no inclusion criteria were applied to pregnant women who participated in the focus group discussion aimed at improving the representation of participants, refining the relevance of attributes for the pilot project, or its subsequent full-scale application.”  (page 22, line 348) |
|  | **Reviewer #2** |  |
|  | This manuscript describes the development of a decision-aid to determine whether additional tests should be added to routine prenatal screening programs. The authors highlight the potentially novel development of decision-criteria that address both pregnant women and policymaker perspectives.  Overall, the manuscript is well organized and generally well-written. While the authors have done an excellent job writing the article, it would benefit from a copy-editor as some grammatical and sentence structures required me to re-read them multiple times to understand what the authors were hoping to convey to the reader (an example is lines 72-75).  All of my comments are relatively minor, but would strengthen the understanding of this work and its contributions to the field. | Thank you very much for the comment.  We solicited scientific editing services from Wiley Editing Services to improve the presentation of the article.  We also paid particular attention to all the sentences pointed out by the reviewers to, hopefully, make them more understandable. |
|  | CONCEPTUAL COMMENTS |  |
|  | 1) Why were you interested in developing a consensus-based model? Understanding differences in perspectives between decision-criteria are as important as addressing similarities. It would be important to highlight the potential limitations (and benefits) of a consensus-based model and how it might shape the buy-in and use of the decision-aid. | - We added the benefit of having a common DCE instrument in the introduction section, as follows:  “Information provided by such a quantitative instrument, which can be administered to a representative sample of stakeholders, is expected to provide evidence of alignments and disagreements between groups of actors, which would complement information coming from qualitative studies.” (page 4, line 59)  - We highlighted limitations of constructing a consensus instrument in the discussion section as follows:  “This study has some limitations. First, about half the individuals who agreed to participate (51%) did not complete the survey. Of these, 78% quit the survey after reading the informed consent form, whereas the rest (22%) stopped answering the first question. The reasons why many potential participants refused to take part in the study were not investigated. However, this observation is commonly reported in DCE studies. The main reasons for the low participation rate presented in the literature are the complexity of the task asked, sensitivity of the study topic, cognitive burden of the DCE questionnaire, and having poor familiarity with trade-off tasks [39, 40].  Second, we were unable to test the instrument with policymakers in the pilot project because of the limited pool of individuals who voted as members of health policy deliberating committees. Indeed, the recruitment of policymakers must consider the fact that their participation would be required not only in the development phase of DCE instrument construction but also in the administration phase of the final version of the DCE questionnaire, which requires a larger sample size. The main consequence of this limitation is that some attributes retained in the final version of the DCE instrument may not be considered essential by policymakers; hence, they may not be statistically significant when administered in a full-scale study.  Third, only primigravidae were included in the interviews. The inclusion of a homogenous population of gravidae in the instrument construction was considered to limit the complexity of the experimental design. We cannot exclude the possibility that subgroups of pregnant women might value instrument attributes differently.  Finally, for practical reasons, only pregnant women between 28-30 weeks’ gestation were included in the testing phase of the study. In addition, these women can be whether or not primigravidae, as required by qualitative studies. This may have affected the results. However, no inclusion criteria were applied to pregnant women who participated in the focus group discussion aimed at improving the representation of participants, refining the relevance of attributes for the pilot project, or its subsequent full-scale application.”  (page 22, line 348)  - Also, we addressed this limitation in the conclusion section, as follows:  “This study showed that it is possible to obtain a consensus list of attributes from pregnant women and policymakers regarding the expansion of NIPS-based programs. Therefore, a single DCE instrument can be constructed to compare preferences between the demand and supply sides of healthcare systems for the eventual addition of new conditions to be screened in the program. However, complete validation of the instrument is limited because there may be too few policymakers to statistically ascertain the relevance of the attributes and their levels.” (page 24, line 407) |
|  | 2) It would be helpful for the authors to describe who they expect the end-users of this tool to be (e.g., whether these types of tools are usually used by policy makers, health care providers, health administrators, families) | We added this information in the conclusion section of the text, as follows:  “Having a common DCE instrument to be administered to beneficiaries of an intervention and policymakers is expected to allow identifying similarities and differences on its value experiences by the two groups. These quantitative data should help policymakers to evaluate whether the perspective of the beneficiaries that they are mandated to consider, is sufficiently taken into account in their recommendations on the provision of an intervention to the population.” (page 24, line 380) |
|  | METHODS AND REPORTING COMMENTS |  |
|  | 1) It would be helpful to describe why the authors chose to work only with primigravidae women. I would think people who may have already had experience with prenatal screening and being parents to children may have different opinions on testing practices. Perhaps the authors could include an overview of any literature available on this subject and/or highlight this as part of the limitations of their work. | We acknowledged this decision as one of study limitation. A paragraph was added in the discussion section as follows:  “Third, only primigravidae were included in the interviews. The inclusion of a homogenous population of gravidae in the instrument construction was considered to limit the complexity of the experimental design. We cannot exclude the possibility that subgroups of pregnant women might value instrument attributes differently.”  (page 23, line 364) |
|  | 2) The authors describe the use of a focus group to determine the selection and framing of attributes (lines 186-191) and state that this was done by email. It would be helpful to describe how a focus group methodology (where interaction between participants and collective discussion plays an important role in the method) was implemented by email. If this was more of a second consultation or modified Delphi process, readers may have an easier understanding how this step was completed. | We described the selection and framing of attributes and levels. This required a 2 steps approach: consultations and focus group discussion. The consultations were done by e-mail. However, the focus group discussion was held physically. All participants were present together in a room at the hospital. Clarification was brought into the methodology section, as follows:  “The focus group was held at the CHUL hospital.” (page 9, line 169)  The authors agreed with the review’s suggestion in describing this phrase as a Delphi process. The modification was made to the methodology section, as follows:  “Attribute selection followed a Delphi process aimed at finding a consensus between both groups of participants on the set of attributes and levels to be included in the DCE instrument [28, 29]. The Delphi approach involved consultations and a focus group discussion. After each step, discussions were held between research team members to refine the list of attributes and levels.” (page 8, line 153) |
|  | 3) It would be helpful for the authors to explain why they placed an inclusion criteria of being between 28-30 weeks gestation (whereas this was not included in development stage). (line 143) | We added a sentence to clarify this criterion in the Methodology section/ Sampling and recruitment of the text, as follows:  “Women enrolled in our study were recruited from a clinical trial on NIPS (PEGASUS-2, ClinicalTrials.gov Identifier: NCT03831256) and agreed to participate in an additional study. The 28th-30th weeks gestational age was chosen to avoid interference with the clinical trial data collection periods.” (page 10, line 200) |
|  | 4) It would be helpful to understand if there was any discussion of re-working the criteria around sensitivity and specificity to make these concepts more accessible to people considering the decision criteria, particularly these were priority considerations of policymakers | We recognized the term “sensibility” as a mistyping error. Correction was made in the text to be “sensitivity”.  No further work was performed on these dimensions because a consensus was considerably reached from both groups throughout several steps:  1. Individual interview  While policymakers raised concern on test sensitivity and test specificity, pregnant women referred these dimensions in more general terms: detection rate/test performance.  2. Consultations  All participants including policymakers and pregnant woman pointed out these are “medical terms might not give much significant meaning to pregnant women and were difficult to understand by a layperson” (line 285).  3. Focus group discussion  The focus group discussion involved pregnant woman and policymaker confirmed the irrelevance of these terms in the questionnaire.  Finally, we discussed this element in the discussion section:  “Moreover, the construction of the DCE instrument shows that a consensus has been reached on a final version, even if some attributes were initially proposed by only one group (test sensitivity and test specificity). This probably reflects the respect and interest of all participants for preoccupation with some attributes that were of little concern to them before the study, but whose evocation aroused interest.”  (page 22, line 338) |
|  | 5) It would be helpful to describe how the cost criteria was described (e.g., was there any mention of who would cover the costs, whether it would be paid for by individual families, by public insurance or other mechanisms) | The “cost related to the test” attribute was presented in the DCE instrument referring to out-of-pocket amount that pregnant women are asked to pay for having the test screened for other anomalies rather than the three common ones (trisomy 21, 18 and 13) which are only funded by healthcare systems in some Canadian provinces (including Québec).  This information was presented in interview guide. We also added clarification to the text, in the methodology section, as follows:  “Depending on the spontaneously produced information, specific questions in the interview guide were asked regarding dimensions that were not discussed. For example, participants were asked how much they would be willing to pay out-of-pocket to undergo test screening for additional conditions rather than the three common ones (trisomy 21, 18, and 13). This question reflects the current situation in Canada, where conditions other than those listed in the prenatal screening program can be screened but at a cost that must be paid out-of-pocket.” (page 7, line 132) |
|  | 6) Lines 217: as someone who does not have expertise in creating DCE tools, it would be helpful for the authors to explain what and/or how this test tells you about how attributes are associated with choices (e.g., the logic or theory behind the analysis) | We provided detail information regarding the analysis in the methodology section. The paragraph is rewritten as follows:  “A DCE study assumes that individuals select the alternative that provides the highest utility (random utility maximization theory) [33]. The preference data were codified in an Excel spreadsheet for analysis using a conditional logit model (SAS, release 9.4; Cary, North Carolina) to estimate the relative importance (utility) of each attribute on the preferences of the participants. The model is expressed as follows:  V = ß0 + ß1condition_1+ ß2test_performance_2+ ß3result_moment_1+ ß4test_certainty_2+ ß5test_sufficiency_2+ ß6info_provided_to_women_1+ ß7info_provided_to_women_2+ß8geographic_2+ß9geographic_3+ ß10cost + ε  where V is the utility derived from a given hypothetical screening test (choice) andεrefers to the error term. All attributes were considered as factors (dummy coded) except for the cost related to the test attribute, which was treated as continuous. Coefficients ß1 to ß10 generated by the regression analysis were given a “+” or “-” to indicate the direction of the preference for each attribute. The significance and size of the coefficients were used to estimate the relative importance of the attributes on choice. The significance level was fixed at 0.25% [34].  (page 11, line 215) |
|  | DISCUSSION COMMENTS |  |
|  | 1) There seems to be a contradiction in the authors' statements on line 250, where they state that a novel element of this DEC tool is that it was reached by consensus, but they they go on to state on lie 257 that they cannot describe if and how consensus was reached on the final attributes. Perhaps this is a wording issue, but would be helpful to re-examine these statements to ensure they are consistent with one another. | The contradiction stated by the reviewer might come from a confusing way of writing. We apologize for it. We rewrote the text to make it clearer the fact that the text referred to 2 studies.  In previously submitted version of the manuscript, line 250 referred to our study, whereas line 257 referred to a published paper.  This paragraph was therefore modified as follows:  “Moreover, the construction of the DCE instrument shows that a consensus has been reached on a final version, even if some attributes were initially proposed by only one group (test sensitivity and test specificity). This probably reflects the respect and interest of all participants for preoccupation with some attributes that were of little concern to them before the study, but whose evocation aroused interest.” (page 22, line 338)  “To our knowledge, only one published DCE instrument in the field of pharmacy subsidy decisions has been administered to both patients and policymakers [38]. However, this study has some uncertainties regarding whether both public and health policymakers/experts were effectively involved in the identification of the questionnaire attributes. Furthermore, this study does not provide information regarding whether or how consensus on the DCE instrument attributes between the two groups has been examined.” (page 22, line 342) |
|  | While the authors did engage both pregnant women and policy makers, it is not clear that there was a rigorous process to build a consensus agreement between them. It seems the authors have prioritized the perspectives of pregnant women (as no policymakers were involved in the piloting, and relatively limited numbers involved in the development, and two of policy makers' priority considerations were excluded from the final tool). | This is a limitation of the study. The text has been rewritten to make this limitation clear. |
|  | In my opinion, this manuscript offers an important first step in bridging these two perspectives, and provides an important perspective in centering patient and family perspectives. However, more needs to be done to ensure that such a tool would be recognized and trusted by the diverse types of decision-makers (policy makers and care providers) who may ultimately be the end-users of such a tool. | Thank you very much for your comments. Hopefully, the clarification and modification made to the manuscript help to improve the presentation of study result. |
| 6 | PLOS authors have the option to publish the peer review history of their article ([what does this mean?](https://journals.plos.org/plosone/s/editorial-and-peer-review-process#loc-peer-review-history)). If published, this will include your full peer review and any attached files.   If you choose “no”, your identity will remain anonymous but your review may still be made public.   Do you want your identity to be public for this peer review? For information about this choice, including consent withdrawal, please see our [Privacy Policy](https://www.plos.org/privacy-policy).  Reviewer #1: No  Reviewer #2: No |  |
