# Supplementary material for: Development of a discrete choice experiment questionnaire to elicit preferences by pregnant women and policymakers for the expansion of non-invasive prenatal screening
Source: PLoS One. 2023 Jun 23;18(6):e0287653. doi: 10.1371/journal.pone.0287653 (PMC10289448; doi:10.1371/journal.pone.0287653)
Supplement: Attachment — Submitted filename: Response to Reviewers 2023.05.10.docx [file pone.0287653.s005.docx]

AUTHORS’ RESPONSE

**Academic Editor’s comments**

| **No.** | **Academic Editor’s comment** | **Authors’ responses** |
| --- | --- | --- |
| 1 | I would insist that you provide more information regarding the results of your literature review. Specifically, I suggest that you include a brief report outlining the key findings in the results section of your paper. This is very important to situate the reader on the origin of the atributes used during the qualitative study. | A brief report of our systematic review of the literature was added, following Editor’s suggestion, to the result section of the paper, as follows:  “Of the 58 DCE publications found during the database search for the systematic review of the literature, 13 met our eligibility criteria (see S1, text for full report). All studies were conducted in high-income countries. While these countries provided their populations with a fully/partially universal health coverage, their jurisdictions varied in terms of patients' financial contribution to prenatal care. Moreover, none of these studies had been conducted with policymakers, i.e., individuals who voted for the offer of prenatal care intervention.  Some attributes appeared quite frequently, notably detection rate (11 studies), risk of miscarriage (nine studies), information content (seven studies) and time in pregnancy when results received (six studies). A cost attribute was included in five DCE studies.  Differences in preferences for different levels of the attributes between woman and other stakeholders (i.e., partners, obstetricians, gynecologists, genetic counselors, clinical geneticists, nurses, and midwife) were presented. Women tended to highly value risk of miscarriage and reception of comprehensive information, while health professionals tended to place higher value on detection rate of the test, moment during pregnancy when test results would become available and capacity to diagnose a condition earlier on. Furthermore, in studies whose DCE questionnaires included a cost attribute, trade-offs were reported between screening options presented to respondents in terms of willingness-to-pay values.” |
| 2 | In addition to the brief report in the results, you can make the unpublished report available as a supplemental file. | We submitted the unpublished full report of the literature review as a supporting file (S1 Text). |
| 3 | Additionally, please ensure that you have completed the appropriate checklist for reporting your literature review. | We submitted the PRISMA checklist for our systematic review of the literature under submission item type: “Other”; Description: “PRISMA checklist”; File name: “PRISMA Checklist.docx”. |

**Reviewers’ comments**

| **No.** | **Reviewers’ Comments to the Author** | **Authors’ responses** |
| --- | --- | --- |
| 1 | Reviewer #2: Many thanks for your responses and for diligently considering each of the reviewers' comments. I appreciated the opportunity to re-read your article. I do still have several comments and suggestions- again all still minor- discussed below. | We thank the reviewer for their comments. Please find below our responses to each point raised by the reviewer. |
| 2 | In the Methods section, while the literature is well-described, the authors state that they completed a systematic review. It would be helpful to know if and hoRw they applied specific review checklists to guide this first portion of their study (e.g., did they follow PRISMA guidelines for a formal systematic reviews? scoping reviews?). Including a description of how the review complied with existing guidelines helps readers have a better understanding of the quality of the findings of the review | A clarification was brought into the method section describing our systematic review of the literature, as follows:  “A systematic review of the literature on the use of DCE in the field of prenatal screening for chromosomal anomalies was conducted by two researchers (HMN and BS), according to the Preferred Reporting Items for Systematic Reviews and Meta-Analyses Protocols 2015 (PRISMA-P 2015) recommendations”  (Line 94)  “As suggested by Soekhai *et al.*, we excluded the search terms “DCE” and “conjoint” since they were reported to yield too many irrelevant results. Attribute terms and their valuation were also used to target DCE conducted in prenatal care”  (Line 105)  “The articles or other documents had to be complete and be in English. There was no restriction of geographical areas applied. Any disagreement regarding the selection of an article was discussed until a consensus was reached. A third researcher (DR), if necessary, was implicated.”  (Line 114)  “As the review of literature aimed primarily at identifying the attributes considered in DCE studies, we considered the 9 first ISPOR items (research questions, attributes and levels, construction of tasks, experimental design, preference elicitation, instrument design, data collection, statistical analysis, results, and conclusions) and not the last one (study presentation).”  (Line 122)  “Data for each retained study were independently extracted and summarized by the two reviewers (MHN and BS). Extraction was about the ISPOR checklist items, to which, one additional item was added: main findings. Any disagreement was solved by discussion and debate. The process of consensus search involved the third researcher (DR).”  (Line 126) |
| 3 | In the Discussion section, the authors state:  The main consequence of this limitation is that some attributes retained in the final version of the DCE instrument may not be considered as essential by some policymakers;, hence, they may not be statistically significant when administered in a full-scale study.  Isn't the inverse also true- that some factors that policymakers may consider important may not be adequately represented in the DCE because there was not fullsome representation (and/or variety of policymakers involved)? I would suggest stating a balanced summary of how limited policymaker participation may have affected study findings. | A sentence was added to the paragraph addressing the inverse effect of this limitation, as follows:  “Moreover, we cannot exclude the possibility that some factors that policymakers may consider important may not be adequately represented in the DCE instrument because of limitation in the variety of policymakers involved.”  (Line 464) |
| 4 | In the Discussion section, Line 144: The women that participated in the testing phase were already participants in a clinical trial on NIPS testing.  Doesn't this also likely makes them a unique group and may be worth mentioning in this same paragraph? | A sentence was added to the paragraph clarifying that pregnant women in the testing phase were randomly selected among participants of a representative sample of the population enrolled in a currently running randomize clinical trial.  The sentence is as follows:  “These women were randomly selected from a representative sample of the population enrolled in an on-going randomized clinical trial.”  (Line 472) |
| 5 | In the conclusion, Line 157, the authors state: This study showed that it is possible to obtain a consensus list of attributes from pregnant women and policymakers regarding the expansion of NIPS-based programs.  I am not convinced that the study described what you could call a consensus.  I refer back to my previous comment that while the authors did engage both pregnant women and policy makers, it is not clear that there was a rigorous process to build a consensus agreement between them. It seems the authors have prioritized the perspectives of pregnant women (as no policymakers were involved in the piloting, and relatively limited numbers involved in the development, and two of policy makers' priority considerations were excluded from the final tool). I would suggest changing this sentence to better align with what the study reported. | A clarification was brought to the results regarding the question of prioritization of women’s perspective by showing that policymakers’ preoccupations were all considered in the final instrument. The addition is as follows:  “The participants suggested only one major change. They agreed that ‘test specificity’ and ‘test sensitivity’ concepts were difficult to understand by pregnant women and expressed in test performance attribute. The test specificity and test sensitivity attributes were therefore redundant.”  (Line 336)  We also agreed with the reviewer that a consensus has not been reached among pregnant women and policymakers in the present study. We are aware that building a consensus is a difficult task because it implies an agreement involving representative samples of the population. We therefore changed the term “consensus” to be “agreement” between the two stakeholders’ representative. The sentence in the conclusion section was rephrased as follows:  “This study showed that the achievement of a consensus agreement from the two groups should be granted with better involvement of policymakers in each step of the DCE instrument construction. Moreover, this study demonstrated a DCE development process that allows to obtain a relative agreement on the list of attributes that could be used to construct a single DCE instrument.”  (Line 495) |
| s | Grammatical comments  Overall, the article is easier to read and more clear. I also applaud the authors for writing in a language that is not necessarily their first language. However, it could still do with some improvements. I pulled out some sentences that stood out to me (and suggested some re-wording). | We thank the reviewer for pointing out these linguistic errors and we appreciate reviewer’s suggestions for modification. Correction was then made. |
|  | - Within the abstract (towards the end): are you missing a word? (‘the test result presented to pregnant women is about’, this is better stated in actual results section as ‘information provided from test result', and could also be better described in table 4) | The correction was made from “the test result presented to pregnant women is about” to be “information provided from test result” across the manuscript. |
|  | - Also in abstract, the final sentence could be clearer (e.g., we did not/were not able to engage enough policymakers rather suggesting that they do not exist) | We did not intend to suggest the inexistence of policymakers. We added a clarification to the sentence emphasizing the fact that the number of members in HTA agencies committees is limited.  “However, complete validation of the consensual instrument is limited because there are too few voting members of health technology assessment agencies committees to statistically ascertain the relevance of the attributes and their levels.”  (Line 19) |
|  | - (methods) The pilot project was also used to confirm the domination choice defined from the qualitative studies that will be added to ain the full-scale study with pregnant women and policymakers, to test the plausibility of the choices made. Perhaps better as: The pilot project confirmed the attributes to be added to the full-scale study where the plausibility of choices will be tested with pregnant women and policymakers | We changed the sentence following reviewer’s suggestion:  “The pilot project confirmed the attributes to be added to the full-scale study where the plausibility of choices will be tested with pregnant women and policymakers.”  (Line 207) |
|  | - (discussion) This step showed that pregnant women and policymakers have many common preoccupations besides their particular ones. Perhaps better as: This step showed that pregnant women and policymakers have both common and unique preoccupations | We acknowledge that the reviewer’s suggestion is more precise. Change was made as follow:  “This step showed that pregnant women and policymakers have both common and unique preoccupations.”  (Line 284) |
|  | - (discussion) This probably reflects the respect and interest of all participants for preoccupation with some attributes that were of little concern to them before the study, but whose evocation has aroused an interest   Perhaps better as: This probably reflects a changing understanding of some attributes that were of little concern before the study, but whose perceived relevance grew over the course of the study. | We changed the sentence following reviewer’s suggestion, as follow:  “This probably reflects a changing understanding of some attributes that were of little concern before the study, but whose perceived relevance grew over the course of the study.”  (Line 436) |
|  | - (line 146) In addition, these women can be whether or not primigravidae (??) Several examples of different tenses being used in the same paragraph | We made correction to the paragraph as follow:  “In addition, participants of this phase might consist of multigravida, rather than only primigravidae as in the qualitative studies. This might affect the study results.”  (Line 474) |
